# Application and Progress of Artificial Intelligence in Fetal Ultrasound

**DOI:** 10.3390/jcm12093298

**Published:** 2023-05-05

**Authors:** Sushan Xiao, Junmin Zhang, Ye Zhu, Zisang Zhang, Haiyan Cao, Mingxing Xie, Li Zhang

**Affiliations:** 1 Department of Ultrasound Medicine, Union Hospital, Tongji Medical College, Huazhong University of Science and Technology, Wuhan 430022, China; 2 Clinical Research Center for Medical Imaging in Hubei Province, Wuhan 430022, China; 3 Hubei Province Key Laboratory of Molecular Imaging, Wuhan 430022, China

**Keywords:** fetal ultrasound, artificial intelligence, prenatal diagnosis, deep learning, convolution neural network

## Abstract

Prenatal ultrasonography is the most crucial imaging modality during pregnancy. However, problems such as high fetal mobility, excessive maternal abdominal wall thickness, and inter-observer variability limit the development of traditional ultrasound in clinical applications. The combination of artificial intelligence (AI) and obstetric ultrasound may help optimize fetal ultrasound examination by shortening the examination time, reducing the physician’s workload, and improving diagnostic accuracy. AI has been successfully applied to automatic fetal ultrasound standard plane detection, biometric parameter measurement, and disease diagnosis to facilitate conventional imaging approaches. In this review, we attempt to thoroughly review the applications and advantages of AI in prenatal fetal ultrasound and discuss the challenges and promises of this new field.

## 1. Introduction

Ultrasound has become the primary tool for prenatal imaging diagnosis with its excellent performance as well as its noninvasive and nonradiative nature, real-time display, convenience, and low cost [1,2,3]. During pregnancy, prenatal ultrasound is the most critical imaging examination because it evaluates the growth status, detects congenital defects, and assists clinicians in diagnosis by imaging the fetus and its appendages. This technology helps doctors quickly intervene in the progression of diseases [4,5]. Regular ultrasound examinations for women during pregnancy can effectively reduce congenital disability rates. However, fetal ultrasound is facing some challenges in the clinical pipeline. Many factors affect the accuracy of the examination, such as high fetal mobility, the excessive abdominal wall thickness of pregnant women, and discrepancies between observers, among other things [6]. Acquiring high-quality standard planes, accurate biomedical measurements, and routine disease diagnosis are time-consuming and laborious for ultrasonographers. Thus, optimizing the prenatal ultrasound examination process alleviates sonographers’ workload and improves clinical efficiency and consistency. In recent years, AI has been gradually applied in the field of fetal ultrasound [7]. The emergence of AI-assisted ultrasound imaging is expected to expedite the prenatal checkup process [4,8].

AI refers to solving problems or acquiring knowledge with computer algorithms similar to human intelligence. Machine learning (ML) is a sub-field of AI that focuses on learning and inducing rules from collected data and making inferences and predictions about new data [9]. Unlike other types of ML, which are highly dependent on data quality and expertise, DL, as a primary branch of machine learning, extracts important features from vast amounts of rough data and is exceptionally efficient at image classification, target detection, and image segmentation. It has recently gained popularity in medical imaging [10]. DL algorithms use a CNN comprising multiple hidden layers to extract key features from limited training samples and achieve high-performance predictions [11,12].

**Figure 1:**
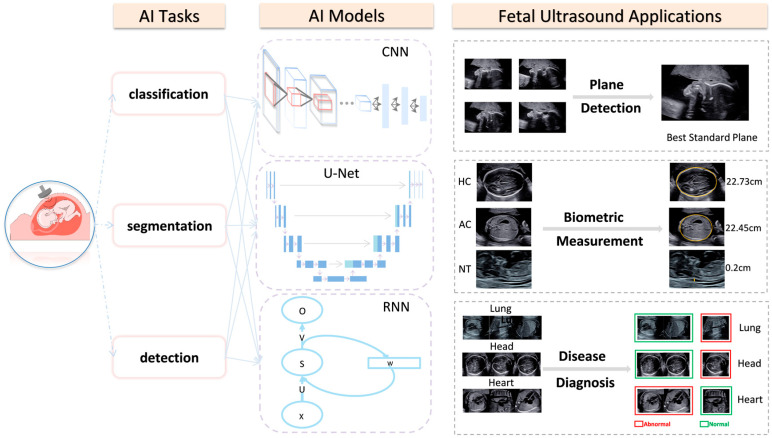
Overview of AI applications in fetal ultrasound. The computer vision tasks involved in developing AI-based fetal ultrasound images are divided into three categories: image classification, image segmentation, and object detection. The figure lists three commonly used AI models in medical images: convolutional neural network (CNN), U-Net, and recurrent neural network (RNN). Current AI applications in fetal ultrasound mainly focus on standard plane detection, biometric measurement, and disease diagnosis.

AI exhibits excellent potential for repetitive ultrasound tasks [8]. The latest ultrasonic imaging equipment has various advanced applications for intelligent imaging. It also reduces novices’ training cycles, improves clinical workflow quality control, and promotes the rational allocation of medical resources [8]. This review provides a systematic overview of the characteristics and application of AI in prenatal fetal ultrasound, concentrating on the automatic recognition of standard views in fetal ultrasound, standardized measurement of biometric parameters, and intelligent disease diagnosis (Figure 1).

## 2. AI Applications in Fetal Ultrasonography

### 2.1. AI Applications in Intelligent Detection of the Fetal Ultrasonic Standard Plane

Fetal standard plane detection relies heavily on the expertise and experience of ultrasound physicians. The main limitation of hand-crafted fetal ultrasound standard plane recognition lies in the high intra-class variability and low inter-class similarity among ultrasound images [13]. In this case, AI can be useful.

Deep convolution neural networks (DCNNs) can distinguish similar ultrasonic views without any manually designed features using their feature representation capabilities. Yu et al. [14] leveraged DCNN to recognize fetal facial standard planes (FFSPs), enhancing clinical pipelines’ recognition performance and optimization. However, the DCNN models were trained with insufficiently labeled samples, which led to overfitting problems and performance degradation. To resolve limited training data and performance decline issues, Chen et al. [15] proposed a transfer learning strategy for implanting the knowledge of specific CNN models, pre-trained on a large scale using natural scene images, to localize medical images such as fetal abdominal standard planes (FASP). The model’s accuracy, precision, recall, and F1 score on testing images were 0.896, 0.714, 0.710, and 0.712, respectively. These measurements confirmed the efficacy of DCNN and transfer learning in the auxiliary task of plane recognition [15]. Due to its reliance on quantitative medical datasets and the corresponding convergence problem, the algorithm could not be manipulated in real time, restricting its clinical applications. The multi-task learning framework proposed by Lin et al. [16] applied a faster regional CNN for view detection and quality evaluation. Moreover, the exact evaluation of ultrasonic planes was ≤0.5 s. Incorporating clinical prior knowledge modules significantly improved the accuracy of anatomical structure detection. With its prominent speed and performance, this network has the potential to assist fetal prenatal ultrasound standard plane acquisition in US examinations.

Since non-negligible domain differences between natural scenes and fetal ultrasound images rendered the model infeasible, Chen et al. [13] used a compound neural network and multi-task learning framework to detect three standard planes in the same architecture. Shared features were learned on different planes, reducing the demand for large datasets and the imbalance between data classes. Consequently, the model algorithm’s generalization ability and plane detection accuracy improved. Furthermore, the algorithm utilized a long-short-term memory network (LSTM) to extract more time-domain information, ensuring the time consistency of plane classification and the accuracy of ultrasonic video classification. It is worth mentioning that the differential CNN [17] proposed by Qu et al. [18] automatically recognized six fetal brain standard planes (FBSPs) with 92.93% accuracy and high computational efficiency. Unlike previous studies in the second trimester of pregnancy, the CNN model was used to identify and classify key frames of fetal heart echocardiography in the first trimester of pregnancy [19].

Numerous studies have indicated that combining AI and prenatal ultrasound can significantly improve the efficacy and accuracy of plane recognition, reduce the variance between different operators, and confirm the consistency and repeatability of plane adoption (Table 1). However, limitations exist. In current research [13,14,16], one of these limitations is that most of the studies only include healthy cases, and the lack of pathological samples hampers model development and clinical applications. Large-scale, diversified, and high-quality clinicopathological databases must be built and incorporated into the future training and verification of AI algorithms.

### 2.2. AI Applications in the Measurement of Fetal Ultrasonic Biometry Parameters

#### 2.2.1. Intelligent Measurement of Fetal Head Circumference (HC)

HC is a significant biometric indicator for evaluating fetal growth and development in prenatal ultrasonography, assessing gestational age (GA) and weight, and identifying fetal abnormalities [20,21]. The accuracy of fetal HC measurement can be affected by partial boundary missing in cranial ultrasound images and interobserver variation. Ultrasound images also suffer from low contrast and artifacts [22]. Consequently, even highly experienced sonographers find the manual measurement of fetal HC time-consuming and challenging.

The accurate and efficient quantification of HC is crucial in prenatal fetal ultrasonography. Foi et al. [23] reconstructed a fetal skull model using a Gaussian difference algorithm. Unlike previous models [24,25], which used image processing operations to maximize image segmentation matching, this study constructed a template image based on ellipse parameters and the calvarial thickness from the observed 2D image without human-machine interaction, allowing the fully automated measurement of HC and image artifact reduction. In addition, this method showed strong robustness even in images of poor quality. With the rising popularity of DL, more studies have been devoted to the segmentation of the fetal head using CNN. Fiorentino et al. were the first to use a regression CNN trained by distance field to delineate the skull curve [26] with a mean absolute error (MAE) of 1.90 (±1.76) mm and a dice similarity coefficient (DSC) of 97.75 (±1.32)%. The model showed potential for automatically quantifying HC in clinical practices. Another study [27] thoroughly combined transformers’ powerful global feature extraction capability and CNN’s local feature extraction to accurately extract complete information from the skull contour without human-computer interaction. It was a one-stage network that achieved precise automatic measurement of fetal HC in 2D ultrasound images. The algorithm detected the skull contour with an average accuracy of 84.45%, a MAE ± std (mm) of 1.97 ± 1.89, and a ME ± std (mm) of 0.11 ± 2.71 for the HC18 dataset without increasing major computational parameters. At present, many studies have surpassed simple HC measurement. Emerging studies have attempted the automated measurement of other biometric parameters such as fetal biparietal diameter (BPD), cerebellar transverse diameter, and occipital frontal diameter [28,29,30]. In addition to two-dimensional (2D) ultrasound popular in clinical practice, three-dimensional (3D) ultrasound has also been adopted to present cubic anatomical structures, providing richer spatial information and quantitative biometric parameters in combination with the hybrid attention scheme (HAS) for the whole fetal head segmentation that are more representative and comprehensive [1].

The combination of conventional HC measurements in ultrasound with AI reduces examination time, inter-clinician variability, and increases diagnostic accuracy [26]. The current direction is to incorporate more and better-quality datasets [1] and design enhanced network structures to improve performance. Smartplanes software [31] can automatically measure HC and BPD in 3D ultrasound with good reproducibility, which has been put into clinical use. We should integrate more algorithms into clinical practice and obtain timely feedback from clinical users to solve clinical problems [30].

#### 2.2.2. Intelligent Measurement of the Fetal Abdominal Circumference (AC)

AC is the principal parameter for calculating fetal weight [32], which holds great clinical value in evaluating fetal growth and early screening for intrauterine growth restriction or oversized fetuses [33]. Improving its measurement accuracy can reduce fetal morbidity and mortality from these diseases. In clinical practice, sonographers must locate the standard plane of the abdomen manually. The variability of fetal posture, oligohydramnios, and pregnant women’s abdominal wall thickness can affect the accuracy of measuring AC [8]. Therefore, a rapid and accurate method of measuring AC is urgently needed to ease the burden on sonographers.

Clinical practice calls for reliable automatic image segmentation of the abdominal circumference. CNN has displayed significant advantages in medical image classification. Jang et al. [32] first used CNN to classify ultrasonic images and then adopted the Hough transform to measure AC automatically. With only a few training samples and some artifacts in the images, the algorithm could still stably measure AC parameters with a DSC of 85.28 ± 10.08%. However, insufficient amniotic fluid in pregnant women may hamper the accuracy of the AI algorithm in predicting AC [32]. Kim et al. [33] proposed an AI algorithm combining multiple CNNs and U-Net [2] to achieve multi-task learning. It accurately identified the fetal abdominal region with the support of fetal rib and spine position information and reduced the influence of amniotic fluid deficiency and artifacts on AC measurement. Similarly, a study combining a multi-scale feature pyramid network and U-Net for image segmentation incorporated an attention gate (AG) into the network, which restrained the feature activation of unrelated regions and boosted the model’s sensitivity and accuracy with a DSC up to 0.98. The automatic multi-parameter measurement of AC, HC, BPD, and femur length strongly correlates with manual methods. Therefore, no additional user intervention is necessary.

Compared with other body parts, abdominal ultrasound images have low and uneven contrast against backgrounds, irregular shapes, high scanning variability, and a blurred edge [33]. Thus, the accurate measurement of AC is especially laborious to perform. AI-aided automated fetal AC measurements can simplify the workflow [34], overcome operators’ dependence [33], and intelligently process artifacts in ultrasound images [32]. Yasutomi et al. [22] confirmed that deep neural networks could be used to estimate the intensity of shadows shown in ultrasound images, which could be used as an image pre-processing step to filter low-quality images.

#### 2.2.3. Intelligent Measurement of Fetal Nuchal Translucency (NT) Thickness

Nuchal translucency (NT) is the fluid-filled area under the skin of the posterior fetal neck [8]. Thickening of the NT can be associated with poor pregnancy outcomes and some chromosomal diseases, such as Down’s syndrome [35,36,37]. NT thickness should be measured in the standard sagittal plane of the fetus for precise measurement, facilitating early detection of fetal structural abnormalities and genetic defects. However, standard plane acquisition and intelligent measurement of NT thickness are difficult to obtain. These challenges include the low signal-to-noise ratio of ultrasound images, the short fetal parietal-rump length, and the mobility of the fetus in early gestation. Unskilled sonographers spend 25.56% more time on crucial biometric tasks than experts [38].

In recent years, multidisciplinary experts have made many breakthroughs in the automatic measurement of NT [8]. Moratalla et al. [39] developed an AI algorithm for semi-automatic measurements of NT that achieved an inter-operator standard deviation of 0.0149 mm, lower than the manual approach of 0.109 mm, significantly reducing inter- and intra-observer differences. Since the semi-automatic approach involves manual fine-tuning of the NT region, which is time-consuming and may lead to interpersonal and intrapersonal variance, some researchers have developed AI algorithms to achieve fully automatic measurements of NT thickness [37,40]. Deng et al. [40] proposed a hierarchical model verified by 690 clinical NT ultrasound images that could simulate the human cognitive process. The model first identified and localized the whole body of the fetus when ultrasound images were shown. Based on the anatomical structure information, it then inferred the NT region and fetal head from images. Identifying the NT region and fetal head improved the model’s accuracy in detecting fetal body anatomy. Compared with the single support vector machine (SVM) classifier, this new model boosted performance by about 5.68% on average, indicating that contextual information facilitates performance. Lastly, the automatic NT measurement proposed by Sciortino et al. [37] did not require user intervention and avoided operator variability. Overall, up to 99.95% of planes were recognized correctly.

AI can assist ultrasonographers in automatically identifying the neck region in ultrasound images and measuring NT. SonoNT [39] has already been integrated into commercial ultrasound equipment that can semi-automatically measure NT in clinical practice. To improve clinician efficiency and examination accuracy, fully automated commercial tools for intelligent NT measurement are expected to be developed.

In summary, the automatic measurement of fetal biological parameters by AI can reduce errors between inter- and intra-operator measurements, promote clinical efficiency, and improve the accuracy of automatic measurement [8], showing a performance level comparable to that of ultrasound experts (Table 2). It is a promising tool for assisting inexperienced junior ultrasonographers in making correct clinical decisions [41]. Development in automatic measurement will benefit advancement in precision medicine and help alleviate the global shortage of prenatal ultrasonographers. However, there are a few pressing issues to address. For instance, more research has emphasized automatic head circumference measurement than abdominal circumference and long bones. We hoped that AI algorithms would fully automate tasks involving simultaneous multi-parameter measurements, which would promote the standardization and automatization of fetal ultrasound examination.

### 2.3. AI Applications in Fetal Ultrasonic Diseases Diagnosis

#### 2.3.1. AI Applications in Fetal Ultrasound of Neonatal Respiratory Diseases

Lung hypoplasia is the most common cause of premature mortality and neonatal respiratory morbidity (NRM) [42]. Clinicians perform biochemical analyses of amniotic fluid by amniocentesis to accurately assess fetal lung maturity (FLM). However, the results may be compromised when amniotic fluid is mixed with meconium or blood. Moreover, the invasive procedure may cause harmful complications. Ultrasound has developed significantly in recent decades as a noninvasive and reproducible method for assessing fetal lung maturity. In conventional ultrasound, there is a good correlation between ultrasound image changes and FLM when comparing the echogenic differences between the fetal lung and placenta, fetal intestine, or liver. This correspondence allows for the noninvasive prediction of FLM [43]. However, various factors, such as instrumentation, subjective examiner variation, and maternal-fetal status, limit its clinical application.

Texture feature analysis can extract key features directly from ultrasound images and effectively quantify FLM, thereby reducing subjective examiner variation. Palacio et al. [43] proposed an automatic quantitative ultrasound analysis (AQUA) texture extractor that could extract the most relevant features from fetal lung ultrasound images for FLM quantification. They achieved FLM prediction with a sensitivity of 95.1%, a specificity of 85.7%, and an accuracy of 90.3%. Based on the former study, Bonet-Carne et al. [44] proposed a new quantitative ultrasound fetal lung maturation analysis method, called quantusFLM, which could predict the occurrence of respiratory distress syndrome in newborns with an accuracy comparable to the amniotic fluid test. More importantly, this model allowed for immediate clinical application. Palacio’s team [45] conducted a prospective study in 20 centers worldwide, using quantusFLM to analyze 730 images. They predicted the incidence of neonatal respiratory distress syndrome with an accuracy of 86.5% and a specificity of 88.6%. Another study [46] applied quantusFLM to twin pregnancy groups. Xia et al. [47] developed a normal fetal lung GA grading model to identify abnormal fetal lung development caused by maternal gestational diseases. The model could also evaluate lung maturity after antenatal corticosteroid (ACS) therapy. The model achieved an overall accuracy of 83.8% in predicting GA, with good stability and reproducibility. Given the strong correlation between GA and FLM, the model showed excellent potential for assessing neonatal respiratory distress syndrome. AI-based technology has provided new ideas for the detection of FLM in fetal ultrasound images.

#### 2.3.2. AI Applications in Fetal Ultrasound of Intracranial Malformations and GA Estimation

- Intracranial malformations

Central nervous system (CNS) malformations are among the most common congenital anomalies, and the incidence of brain abnormalities may be as high as 1% [48]. Currently, clinical diagnosis of brain ultrasound-suspected brain abnormalities may be adjusted or improved by amniocentesis or MRI findings. However, the former is invasive with a risk of post-puncture complications [49], and the latter is susceptible to fetal movement, so it cannot capture tiny cysts. As a noninvasive, radiation-free, real-time, and dynamic imaging technique, fetal neurosonography (NSG) has unique advantages in diagnosing fetal central system disorders. However, sonographers identify fetal brain planes manually in clinical practice. Incorrect fetal head position, maternal obesity, and a lack of expertise and experience can affect imaging quality and final diagnostic results, contributing to high false-positive and false-negative rates.

AI-assisted ultrasound diagnosis can help overcome the limitations of traditional ultrasound examination. Xie et al. [50] advanced the first algorithm for prenatal ultrasound diagnosis of fetal brain abnormalities. This model used U-Net to segment cranial regions and the VGG-Net network to distinguish normal and abnormal ultrasound images, helping reduce the false-negative rate of fetal brain abnormalities. Although the accuracy of lesion region localization was low, it could be compensated by object detection techniques [51] or back-propagated approaches. Xie et al. [52] used a CNN-based DL model to distinguish normal and abnormal fetal brains with an overall accuracy of 96.31%. Furthermore, the model could visualize the lesion site through heat maps and overlapping images, which boosted the sensitivity of the essential clinical examination. However, both studies could only distinguish normal from abnormal standard brain planes. Based on the YOLO algorithm, Lin et al. [53] developed and validated an AI-assisted image recognition system, PAICS, which could detect and classify nine kinds of fetal brain malformations in real time. The model required less time, and its performance was comparable to that of experts. Due to its significant progress in this field, AI is expected to become an effective tool for clinically screening fetal CNS malformations with improved prenatal detection rates.

- GA estimation

Another important application of AI combined with fetal brain ultrasound is GA estimation. Currently, ultrasound measurements of fetal anatomical landmarks have been well established for GA estimation, especially in early gestational states. However, with time, the error in ultrasound-estimated GA becomes more pronounced in late pregnancy due to the neglect of variability in fetal growth and development, and in some studies, the error is greater than 2 weeks [54,55]. Therefore, the development of an accurate and reliable model for mid- and late-stage GA assessment is worth exploring.

Namburete et al. [56] used the regression forest method to analyze the spatial and temporal association between brain maturation and GA in fetal cranial ultrasound images. The estimated GA was close to the value obtained by clinical measurement, with the root mean square error (RMSE) of ±6.10 days in the second and third trimesters. The team developed a feature selection framework based on 448 3D ultrasound images of the fetal brain that was able to identify the key anatomical regions of the brain associated with GA changes, including callosal sulci, the Sylvian fissure, and the cingulate [56]. Additionally, Burgos-Artizzu et al. [57] proposed a new DL model named quantusGA based on standard transthalamic axial plane 2D images of 1394 fetuses. The method used supervised learning to learn and automatically analyze changes in brain morphology in fetal ultrasound images. This method [57] showed a lower error in late pregnancy than simply measuring fetal biometric parameters. Unlike single image analysis [56,57], Lee et al. [55] used CNN to analyze images from multiple standard ultrasound views for GA estimation without utilizing biometric information. The best model has a MAE of 3.0 days and 4.3 days in the middle and late stages of pregnancy, respectively. What’s more, it’s applicable to both high- and low-risk pregnancies and to people in different geographical areas. The application of AI has the potential to provide a reliable and accurate GA prediction method for pregnant women who are unable to attend early obstetric examinations in a timely manner.

#### 2.3.3. AI Applications in Fetal Ultrasound of Congenital Heart Diseases

Congenital heart disease (CHD) is the most common and severe congenital disease among newborns, with a prevalence of about 6–13 per 1000 [29,58]. Rates of fetal congenital heart disability have reached 9.3% in Asia [59]. Generally, surgical treatment for neonatal and adult CHD patients is costly, with long treatment cycles, the risk of secondary surgery, and high mortality, placing a heavy burden on patients and their families. Prenatal ultrasound diagnosis of fetal CHD can assist in making clinical decisions and improve neonatal outcomes [19]. However, when identifying complex abnormal fetal heart anatomy [60,61], detecting and localizing lesions precisely is difficult and time-consuming due to the activity of the fetus, the faster heart beating, the smaller heart size than adults, and the high requirement for expertise [62,63,64]. Moreover, in countries or regions lacking well-established healthcare systems, advanced echocardiographic equipment, and experienced technicians or specialists, prenatal CHD has a high rate of missed diagnosis, which can lead to delayed treatment and a poorer prognosis. The combination of AI and traditional ultrasound is expected to alleviate the above problems [62,65].

In recent years, AI techniques have made significant progress in assessing cardiac structure and function. Arnaout et al. [5] trained an integrated neural network model based on 1326 2D ultrasound grayscale images to distinguish normal hearts from complex CHDs in the recommended five standard cardiac views (three-vessel trachea, three-vessel view, left ventricular outflow tract, axial four-chamber, and abdomen). The model was tested internally with a concentration AUC of 0.99, a sensitivity of 95% (95% confidence interval, 84–99%), a specificity of 96% (95% confidence interval, 95–97%), and a negative predictive value of 100%. Their results showed that the model’s sensitivity is comparable to that of clinicians and performs well on both external datasets and lower-quality images. Compared to 2D ultrasound, four-dimensional (4D) sonography with spatiotemporal image correlation (STIC) enables a more comprehensive view of fetal malformations in real time. Yeo et al. [60,61] developed a fetal intelligent navigation echocardiogram (FINE) in conjunction with Virtual Intelligent Sonographer Assistance (VIS-Assistance^®^), allowing clinicians to locate seven anatomical landmarks according to prompts. Seconds later, the software could automatically generate nine standard fetal echocardiographic views and intelligently identify surrounding anatomical structures with Vis-Assistance. In four proven cases of CHD (coarctation of the aorta, tetralogy of Fallot, transposition of the great vessels, and pulmonary atresia with an intact ventricular septum), the FINE model could recognize abnormal cardiac anatomy. This approach may simplify fetal heart examinations and reduce operator dependence. In a follow-up study, FINE further identified double-outlet right ventricle (DORV) [66] and d-transposition of the great arteries (d-TGA) [67]. It has been integrated into commercial ultrasound equipment [68]. Based on earlier studies [60,61], Yeo et al. [69] proposed a model combined with color or bidirectional functional Doppler, namely 5D Heart Color (or color Doppler FINE). In four specific CHD cases, 5D Heart Color showed vascular anatomy, flow direction, and velocity, providing additional diagnostic details differentiating CHD from micro-physiological tricuspid regurgitation and improving diagnostic accuracy and sensitivity. However, the visualization rate was low for the superior and inferior vena cavae views (33 and 30%), and the STIC technique has a high requirement for the examination equipment and extra time costs [70]. Anda et al. proposed the use of learning deep architectures for the interpretation of first-trimester fetal echocardiography (LIFE) to recognize fetal CHD without using 4D sonography, which was the first AI-standardized approach to assist sonographers in diagnosing fetal CHD in the first trimester [70]. AI has shown significant clinical potential in congenital disease diagnosis, shortening training periods, and reducing the subjective variability of clinicians [71] (Table 3).

Gong et al. [59] proposed a new model named DGACNN, which could achieve a recognition rate of 85% for fetal CHD, even better than that of experts.

However, compared with neonatal and adult studies, fetal ultrasound image quality can be affected by various factors, such as maternal abdominal fat, fetal position, and mobility [8]. These factors limit the intellectual development and clinical applications of prenatal ultrasound diagnosis. Therefore, further exploration is needed.

## 3. Limitations and Future Perspectives

In this paper, we review AI applications in identifying prenatal ultrasound views, automated measurement of biological parameters, and disease diagnosis. Not only does AI significantly improve clinical efficiency, but it also helps to reduce subjective variability due to differences in operator expertise and to standardize plane acquisition [41]. Moreover, it provides potential solutions for areas with scarce medical resources. However, limitations remain, and more research is needed before AI-assisted ultrasound imaging diagnosis can realize its full potential. Firstly, current studies focus more on the algorithm than on clinical utility. Insufficient algorithms can eventually be transformed into clinical practice. Secondly, most data sets lack pathological cases and only include healthy fetuses and pregnant women. The uneven training data sets result in poor algorithm training performance. Besides, single-source data limits model generalization. Thirdly, most models use supervised learning algorithms that require quantitative manual data labels.

To establish a powerful AI-assisted ultrasound model, multicenter and diversified data should be incorporated into future research. In addition, data quality control standards must be established to ensure the datasets’ quality. As microlesions and complex congenital malformations are difficult to diagnose in real clinical scenarios, more cases of complex and rare diseases must be collected to improve detection rates. On the other hand, as long as model performance can be guaranteed, reducing network complexity and operation volume is desirable. Lightweight AI models such as QF-MobileNetcan [74] and MobileUNet [75] have been designed for clinical diagnosis and treatment.

Moreover, medical ethics are critical in conducting clinical research and may affect the development and application of AI models. Therefore, the diagnostic process should consider questions such as who bears the possible medical risk. In addition, an authentic clinical consultation contains multidimensional information, such as the pregnant woman’s age, GA, and medical history. Multidisciplinary collaboration can facilitate the synthesis of multidimensional information for designing more comprehensive AI models, developing intelligent ultrasound imaging, and achieving better clinical applications. Thus, interdisciplinary communication between AI developers and sonographers must be further strengthened in the future.

## Figures and Tables

**Table 1:** Studies of AI applications in the intelligent detection of fetal ultrasonic standard plane.

Paper	Plane	AI Task	Technology	GA	Dataset	Performance Metric
Chen, H. et al. [13]	FASP FFASP FFVSP	detection	CNN; RNN; LSTM; multi-task learning; 2D US	18–40 w	Training Dataset: FASP(11,942); FFASP(13,091); FFVSP(12,343); Test Dataset: FASP(8718); FFASP(2278); FFVSP(2252)	Accuracy: 0.941; 0.717; 0.846; Precision: 0.945; 0.737; 0.898; Recall: 0.995; 0.955; 0.936; F1score: 0.969; 0.832; 0.917 (FASP; FFASP; FFVSP)
Yu, Z. et al. [14]	FFSP	detection classification	DCNN; transfer learning; 2D US	20–36 w	Training Dataset: 4849; Test Dataset: 2418	Accuracy: 0.9653; Precision: 0.9698; Recall: 0.9700; F1score: 0.9699; AUC: 0.99
Chen, H. et al. [15]	FASP	detection classification	CNN; transfer learning; Barnes-Hut-SNE; 2D US	18–40 w	Training Dataset: 11,942; Test Dataset: 8718	Accuracy: 0.896; Precision: 0.714; Recall: 0.710; F1 score: 0.712
Lin, Z. et al. [16]	FHSP	detection classification	MF R-CNN; transfer learning; 2D US	14–28 w	Training Dataset: 1451; Test Dataset: 320	Accuracy: 0.9625; Precision: 0.9776; F1score: 0.9568; AUC: 0.9889 (From Group B1)
Qu, R. et al. [17]	FBSP	detection	differential-CNN; 2D US	16–34 w	Training Dataset1: 18,000; Training Dataset2: 720; Test Dataset1: 6000; Test Dataset2: 240	Accuracy: 0.910; 0.891; Precision: 0.855; 0.853; Recall: 0.901; 0.864; F1 score: 0.900; 0.901 (Data Set1; Data Set2)
Qu, R. et al. [18]	FBSP	detection classification	DCNN; transfer learning; 2D US	16–34 w	Training Dataset: 18,000; Test Dataset: 6000	Accuracy: 0.9311; Precision: 0.9262; Recall: 0.9239; F1score: 0.9353; AUC: 0.937
Stoean, R. et al. [19]	FECG	detection classification	CNN; DenseNet-201; Inception-V4; ResNet-152; ResNet-18; ResNet-50; Xception; 2D US	12–14 w	Training Dataset: 4260; Validation Dataset: 1495; Test Dataset: 1496	Accuracy: 95%; F1score: 0.9091–0.9958

FASP, fetal abdominal standard plane; FFASP, fetal face axial standard plane; FFVSP, fetal four-chamber view stand and plane; CNN, convolution neural network; RNN, recurrent neural network; LSTM, long-short-term memory network; DCNN, deep convolution neural networks; AUC, area under the receiver operating characteristic curve; FFSP, fetal facial standard plane; FHSP, fetal head standard plane; FBSP, fetal brain standard plane; FECG, fetal echocardiography; MF R-CNN, multi-task faster regional convolutional neural network; mAP, mean average precision; SVM, support vector machine; 2D, two-dimensional; GA, gestational age; US, ultrasound; w, weeks.

**Table 2:** Studies of AI applications in the measurement of fetal ultrasonic biometry parameters.

Paper	Biometry	AI Tasks	Technology	GA	Samples	Performance Metrics
Li, J. et al. [20]	HC	detection classification	ElliFit; random forest; prior knowledge; 2D US	18–33 w	training: 524 images; testing: 145 images	DSC (%): 96.66 ± 3.15; MAE (mm): 1.7; MSD (mm): 1.78 ± 1.58; RMSD (mm): 1.77 ± 1.37; Precision (%): 96.84 ± 2.99
Sobhaninia, Z. et al. [21]	HC	segmentation	multi-task deep CNN; 2D US	12–35 w	8823 images	DSC (%): 96.84; ADF (mm): 2.12; HD (mm): 1.72
Foi, A. et al. [23]	HC BPD OFD	segmentation	DoGEll;multistart Nelder–Mead; 2D US	21, 28 and 33 w	90 images (90 fetuses)	DSC (%): 97.73; RMSE (mm): 4.39 (HC)
Fiorentino, M. C. et al. [26]	HC	segmentation	region-proposal CNN; 2D US	12–35 w	HC18dataset training: 999 images; testing: 335 images	DSC (%): 97.75 ± 1.32; MAD (mm): 1.90 ± 1.76
Yang, C. et al. [27]	HC	detection classification	ResDCN; transformer; SSR; rotating ellipse; KLD loss; 2D US	12–35 w	HC18dataset training: 999 images; testing: 335 images	AP (%): 84.45; MAE ± std (mm): 1.97 ± 1.89; ME ± std (mm): 0.11 ± 2.71
Pluym, I. D. et al. [28]	BPD HC TCD CM Vp	detection	SonoCNS; computer-aided analysis; 3D US	18–22.6 w	143 subjects	The ICC reflected moderate reliability (>0.68) for BPD and HC and poor reliability (<0.31) for TCD, CM, and Vp.
Chen, X. et al. [29]	LV	detection classification segmentation	Mask R-CNN; FPN; RPN; clinical prior knowledge; 2D US	Not reported	training: 2400 images testing: 500 images	MAE (mm): 1.8; SD (mm): 3.4; RMSE (mm): 2.38
Li, P. et al. [30]	HC BPD OFD	segmentation prediction	FCNN; Feature pyramid; ROI pooling; 2D US	12–35 w	HC18dataset training: 999 images; testing: 335 images	DSC (%): 97.94 ± 1.34; ADF (mm): 1.81 ± 1.69; HD (mm): 1.22 ± 0.77
Ambroise Grandjean, G. et al. [31]	HC BPD	detection	Smartplanes 3D US	17–29w	30 subjects	Intra- and interobserver reproducibility rates were high with ICC values >0.98
Yang, X. et al. [1]	fetal head volume	segmentation	HAS; U-net; 3D US	20–31 w	traning: 50 volumes testing: 50 volumes	DSC (%): 96.05; MSD (ml): 11.524
Jang, J. et al. [32]	AC	classification segmentation	CNN; Hough transform; 2D US	20–34 w	training: 56 subjects testing: 32 subjects	DSC (%): 85.28; Accuracy: 0.809 (expert 1); Accuracy: 0.771 (expert 2); Accuracy: 0.905 (between the two experts)
Kim, B. et al. [33]	AC	detection classification segmentation	CNN; U-Net; 2D US	Not reported	training: 112 images testing: 77 subjects	DSC (%): 92.55 ± 0.83; Accuracy (%): 87.10
Ghelich Oghli, M. et al. [2]	BPD HC AC FL	segmentation	CNN; MFP-Unet; AG; 2D US	14–26 w	HC18dataset: training: 999 images; testing: 335 images local dataset: 473 images	DSC (%): 98; HD (mm): 1.14; Conformity: 0.95; APD (mm): 0.2
Moratalla, J. et al. [39]	NT	detection segmentation	semi-automated method; 2D US	11–13 w	48 images (12subjects)	Within-operator SD (mm): 0.05 (semi-automated method); 0.126 (manual method); ICC: 0.98 (semi-automated method); 0.85 (manual method)
Deng, Y. et al. [40]	NT	detection segmentation	hierarchical model; Gaussian pyramids; 2D US	11–13.6 w	690 images (training: 345 images; testing: 345 images)	The spatial model increases the performance by about 5.68% on average compared with the single SVM classifier for the NT in the proposed model
Sciortino, G. et al. [37]	NT	detection segmentation	wavelet analysis; multi-resolution analysis; 2D US	11–13 w	382 images (12 subjects)	True positive rate (%): 99.95

HC, head circumference; DSC, dice similarity coefficient; MAE, mean absolute error; MSD, maximum symmetric contour distance; RMSD, root mean square symmetric contour distance; ADF, absolute difference in head circumference; HD, Hausdorff distance; BPD, biparietal diameter; OFD, occipitofrontal diameter; FCNN, fully convolutional neural networks; RMSE, root mean square error; MAD, mean absolute difference; AP, average precision; ME, mean error; TCD, transcerebellar diameter; CM, cisterna magna; Vp, posterior horn of the lateral ventricle; LV, left ventricular; SD, standard deviation; AC, abdominal circumference; FL, femur length; NT, nuchal translucency; CNS, central nervous system; MFP, multi-feature pyramid; ICC, intraclass correlation coefficient; APD, average perpendicular distance; 3D, three-dimensional; SSR, soft stage-wise regression; KLD, Kullback-Leibler Divergence; FPN, Feature Pyramid Networks; RPN, Region Proposal Network; HAS, hybrid attention scheme; AG, attention gate; GA, gestational age; w, weeks.

**Table 3:** Studies of AI applications in fetal ultrasonic disease diagnosis.

Paper	Organ	Samples	GA	Task	Technology	Performance Metrics
Palacio et al. [43]	Lung	103 subjects	24–41 w	classification prediction	AQUA; genetic algorithm; SVM; 2D US	Accuracy: 90.3%; Sensitivity: 95.1%; Specificity: 85.7%
Bonet-Carne et al. [44]	Lung	>13,000 non-clinical images 957 fetal lung images	28–39 w	classification prediction	quantusFLM; regression model; classification tree; neural network; 2D US	Sensitivity: 86.2%; Specificity: 87%
Palacio et al. [45]	Lung	730 images	25–38.6 w	classification prediction	quantusFLM; regression model; classification tree; neural network; 2D US	Accuracy: 86.5%; Sensitivity: 74.3%; Specificity: 88.6%
Moreno-Espinosa et al. [46]	Lung	262 images 131 pairs of twins	26–38.6 w	classification prediction	quantusFLM; regression model; classification tree; neural network; 2D US	Concordance in the risk of NRM: 97.4%; 73.5%; 88.4% (Group 1; Group 2; Group 3)
Xia et al. [47]	Lung	7013 images 1023 subjects	20–41.6 w	classification prediction	CNN; DenseNet; AlexNet; 2D US	Accuracy: 83.8%; Sensitivity: 91.7%; 69.8%; 86.4%; Specificity: 76.8%; 90%; 83.1%; AUC: 0.982; 0.907; 0.960 (class I; class II; class III)
Xie, B. et al. [50]	Brain	Segmentation Dataset: 13,350 images; Classification Dataset: 11,645 images	18–32 w	segmentation classification prediction	DCNN; U-Net; VGG-net; ImageNet; Grad-CAM; 2D US	DSC: 0.942; F1 score: 0.96
Xie, H.N. et al. [52]	Brain	Normal: 15,372 images, 10,251 subjects; Abnormal: 14,047 images, 2529 subjects	Average normal: 22.4 w; abnormal: 26.3 w	classification prediction	CNN; Keras; 2D US	Accuracy: 96.3%; Sensitivity: 96.9%; Specificity: 95.9%; AUC: 0.989
Lin, M. et al. [53]	Brain	43,890 images, 16,463 subjects	18–40 w	segmentation classification prediction	PAICS; CNN; YOLOv3; 2D US	Internal dataset: mean accuracy: 0.992; external dataset: macroaverage accuracy: 0.963; microaverage accuracy: 0.963
Lee, L.H. et al. [55]	Brain	INTERGROWTH-21st dataset [72]; INTERBIO-21st dataset [73]	/	prediction	ML; CNN; ResNet-50; 2D US	MAE: 3.0 days (2nd trimester) MAE: 4.3 days (3rd trimester) (from the best-performing model)
Namburete, A.I. et al. [56]	Brain	INTERGROWTH-21st dataset [72]; INTERBIO-21st dataset [73]	/	prediction	3D cranial parametrization regression forest 3D US	RMSE: 5.18 days (2nd trimester); RMSE: 7.77 days (3rd trimester)
Burgos-Artizzu, X.P. et al. [57]	Brain	1394 subjects	/	prediction	DL; supervised learning; 2D US	Avg error: 3.03 days (2nd trimester); Avg error: 7.06 days (3rd trimester)
Yeo, L. & Romero, R. et al. [60]	Heart	training: 918 images; 51 STIC volumes; testing: 900 images; 50 STIC volumes	training: 19.5–39.3 w; test: 18.6–37.2 w	detection classification prediction	FINE; VIS-Assistance; STICLoop; 2D US; 4D US	FINE generated nine fetal echocardiography views in 76–100% of cases using diagnostic planes, 98–100% using VIS-Assistance, and 98–100% using a combination of diagnostic planes and/or VIS-Assistance.
Garcia, M. et al. [61]	Heart	2700 images; 150 STIC volumes; 150 subjects	19–30 w	detection classification prediction	FINE; VIS-Assistance; STICLoop; 2D US; 4D US	The success rate of obtaining the four-chamber view, left ventricular outflow tract view, short-axis view of the great vessels/right ventricular outflow tract, and abdomen view was 95% (n = 143) using diagnostic planes and 100% (n = 150) using VIS-Assistance.
Arnaout, R. et al. [5]	Heart	107,823 images; 1326 subjects	18–24 w	segmentation classification prediction	DL; Grad-CAM; Saliency mapping; 2D US	AUC: 0.99; Sensitivity: 95%; Specificity: 96%; Negative predictive value: 100%
Ma, M. et al. [66]	Heart	25 STIC volumes; 25 subjects	15–35 w	detection classification prediction	FINE; VIS-Assistance; 4D US	Display rates (3VT, LVOT, RVOT): 84%, 76%, 84%.
Huang, C. et al. [67]	Heart	28 STIC volumes; 28 subjects	22–37 w	detection classification prediction	FINE; VIS-Assistance; 4D US	FINE successfully showed an abnormal 3VT view in 85.7% (n = 25) of d-TGA cases, 75% (n = 21) for LVOT, and 89.2% for RVOT. The interobserver ICCs in this study were greater than 0.81.
Yeo, L. & Romero, R. et al. [69]	Heart	1418 images; 60 STIC volumes	21–27.5 w	detection classification prediction	FINE; VIS-Assistance; STICLoop; S-flow Doppler; 2D US; 4D US	Color Doppler FINE generated nine fetal echocardiography views (grayscale) using (1) diagnostic planes in 73–100% of cases, (2) VIS-Assistance in 100% of cases, and (3) a combination of diagnostic planes and/or VIS-Assistance in 100% of cases.
Gong et al. [59]	Heart	3596 images	18–39 w	recognition classification	DANomaly; GACNN (Wgan-GP and CNN); generative adversarial network; transfer learning; 2D US	Accuracy: 0.850; AUC: 0.881
Anda, U. et al. [70]	Heart	≥6000 images	12–13.6 w	detection classification	CNN	The IS can assist the early-stage sonographers in helping and training for accurate detection of the four first-trimester cardiac key-planes (four-chamber view, left and right ventricular outflow tracts, three vessels, and trachea view).

AQUA, automatic quantitative ultrasound analysis; NRM, neonatal respiratory morbidity; CNN, convolutional neural network; AUC, area under the receiver operating characteristic curve; DSC, dice similarity coefficient; CI, confidence interval; PAICS, prenatal ultrasound diagnosis AI conduct system; FINE, fetal intelligent navigation echocardiogram; DL, deep learning; Grad-CAM, Gradient-weighted Class Activation Mapping; ICC, intraclass correlation coefficient; FLM, fetal lung maturity; STIC, spatio-temporal image correlation; SVM, support vector machine; YOLOv3, You Only Look Once, version 3; GA, gestational age; 4D, four-dimensional; w, weeks; IS, Intelligent Decision Support System; Avg error: average absolute error; MAE, mean absolute error; RMSE, root mean square error.

## Data Availability

Not applicable.
